# Current News

**Published:** 1898-11

**Authors:** 


### Current News.

### SOUTHERN CALIFORNIA DENTAL ASSOCIATION.

The meeting was called to order at 2.30 p.m., the President, Dr. W. A. Smith, in the chair.

The address of welcome was delivered by Dr. Emma T. Read, San Diego.

This was followed by the president's address.

The following papers were read:

"How I fill Root-Canals with Bibulous Paper Points." By Dr. E. W. Sheriff, San Diego.

"Peridental Inflammation." By Dr. Edgar Palmer, Los Angeles.

"Relation of Dentistry to Medicine." By P. C. Remondino, M.D., San Diego.

"Dental Education." By Dr. H. R. Harbison, San Diego.

"Dental Education." By Dr. E. L. Townsend, Los Angeles.

### CLINICS.

Quick mode of gold and silver plating for regulating appliances. Dr. H. R. Harbison, San Diego.

Preparing casts with aluminum as a lining for rubber plates. Dr. J. A. Cronkhite, Los Angeles.

Anchorage in Orthodontia. By Dr. D. R. Wilder, Los Angeles. This was in the form of a talk, the doctor using the black-board to illustrate his method.

Dr. George H. Cushing, formerly of Chicago, was elected to honorary membership.

This being the first annual meeting, over fifty members of the profession signed the membership roll.

It was the largest dental meeting ever held in Southern California, and speaks well for the society's future.

ELECTION OF OFFICERS.

All of the old officers were re-elected, as follows:

President, W. A. Smith, D.D.S., Los Angeles; First Vice-President, H. R. Harbison, D.D.S., San Diego; Second Vice-President, Dr. C. W. Sylvester, Riverside; Treasurer, Dr. J. M. White, Los Angeles; Secretary, L. E. Ford, D.D.S., Los Angeles.

The meeting adjourned to meet in Los Angeles the first Tuesday in October, 1899.

L. E. Ford, D.D.S.,
*Secretary.*

